# Targeted versus universal prevention. a resource allocation model to prioritize cardiovascular prevention

**DOI:** 10.1186/1478-7547-9-14

**Published:** 2011-10-06

**Authors:** Talitha L Feenstra, Pieter M van Baal, Monique O Jacobs-van der Bruggen, Rudolf T Hoogenveen, Geert-Jan Kommer, Caroline A Baan

**Affiliations:** 1 Centre for Prevention and Health Services Research, National Institute for Public Health and the Environment (RIVM), Bilthoven, the Netherlands; 2 Department of Epidemiology, University Medical Centre Groningen, Groningen, the Netherlands; 3 Institute for Medical Technology Assessment, Erasmus University Rotterdam, Rotterdam, the Netherlands; 4 Expertise Centre for Methodology and Information Services, RIVM, Bilthoven, The Netherlands; 5 Centre for Public Health Forecasting, RIVM, Bilthoven, the Netherlands; 6 EMGO Institute for Health and Care Research, VU University Amsterdam, Amsterdam, the Netherlands

## Abstract

**Background:**

Diabetes mellitus brings an increased risk for cardiovascular complications and patients profit from prevention. This prevention also suits the general population. The question arises what is a better strategy: target the general population or diabetes patients.

**Methods:**

A mathematical programming model was developed to calculate optimal allocations for the Dutch population of the following interventions: smoking cessation support, diet and exercise to reduce overweight, statins, and medication to reduce blood pressure. Outcomes were total lifetime health care costs and QALYs. Budget sizes were varied and the division of resources between the general population and diabetes patients was assessed.

**Results:**

Full implementation of all interventions resulted in a gain of 560,000 QALY at a cost of €640 per capita, about €12,900 per QALY on average. The large majority of these QALY gains could be obtained at incremental costs below €20,000 per QALY. Low or high budgets (below €9 or above €100 per capita) were predominantly spent in the general population. Moderate budgets were mostly spent in diabetes patients.

**Conclusions:**

Major health gains can be realized efficiently by offering prevention to both the general and the diabetic population. However, a priori setting a specific distribution of resources is suboptimal. Resource allocation models allow accounting for capacity constraints and program size in addition to efficiency.

## Background

Lifestyle risk factors, especially a high body weight, play an important role in the development of diabetes [1,2]. Due to ongoing ageing and unfavourable trends in lifestyle in the population diabetes prevalence is increasing rapidly [3,4]. Diabetes patients risk a number of micro and macro vascular complications, with 40 to 56% of the patients suffering from one or more of these. Macrovascular complications are responsible for the majority of complication related use of health care and consist of cardiovascular disease and stroke [5]. Prevention aiming at the reduction of cardiovascular risks has therefore the potential to reduce the burden of diabetes [6,7] and is included in current diabetes guidelines. However, given the prevalence of cardiovascular disease in the general population, it seems also worthwhile to introduce similar prevention measures for a broader public [8]. The question thus arises what would be the best strategy: to target cardiovascular prevention to diabetes patients, to invest in prevention strategies intended for the general population, or doing a mix of both?

Part of the answer to this question depends on the relative efficiency of prevention in the general population versus prevention targeting the high risk group of diabetes patients. Numbers needed to treat are lower in diabetes patients, but intervention costs and effectiveness may differ.

Economic evaluations for a range of lifestyle and drug interventions targeting diabetes patients,[9,10] or the general population [11-15] have been published in recent years. Evaluations of drug interventions dominate, but smoking cessation and overweight reduction have also been evaluated frequently. The majority of these evaluations applied some form of modelling to extrapolate from the short term effects on intermediate outcomes such as quitting smoking, weight reduction and lowering cholesterol levels to the outcome of interest: long term health in terms of mortality and quality of life. Trials with a follow-up long enough to directly measure these outcomes are rare, with the notable exception of the UKPDS [16]. Modelling has the added advantage that several sources may be combined to provide a consistent picture of the best available evidence [17].

However, comparing the outcomes of single evaluations is difficult, among others since they were performed in different countries [18,19]. Furthermore, not all evaluations included all relevant effects of the interventions. Comparability is importantly increased when all interventions are evaluated with the same model in the same setting. Therefore, in this paper, we translated evidence for all interventions to a single setting (that of the Dutch healthcare system) and evaluated them using the same model. This model was developed to capture all relevant health effects of the types of prevention that were evaluated, that is, not only effects on cardiovascular diseases, but also those on other chronic diseases that show increased risks for the risk factors targeted by the preventive interventions. Furthermore, effects of prevention on delaying mortality leading to diseases and costs of care in life years gained were also taken into account [20,21]. This improved the comparability of the outcomes and allowed to analyze the full trade-off between different target groups for prevention.

We show that such a comparison, however, cannot be restricted to cost-effectiveness ratios. While informative, it is clear that a prevention program for the general population with a potential reach of 300,000 people will be valued differently from a program fit for a selective patient group consisting of 30,000 people. In other words, program sizes matter [22].

Mathematical programming models for resource allocation combine the results of cost effectiveness analysis with epidemiological and demographic data, as well as data on program scale to find the optimal allocation of resources over programs. Compared to a cost-effectiveness analysis, the strength of a mathematical programming approach is that program sizes and hence budgetary impact are taken into account. The resulting choices of interventions are different from those guided by cost-effectiveness only.

The approach furthermore allows analyzing the effect of different objectives and constraints, for instance on indivisible programs or equity [23,24]. Resource allocation models for diabetes or its prevention have been undertaken previously [23,25,26]. These studies focused on either primary prevention in the non diabetes population or on prevention of complications for diabetes patients separately. In contrast, the current study aimed to compare both, and used resource allocation modelling to address choices between both types of prevention, considering a range of prevention programs and evaluating them using a model that accounts for the full effects on health and costs of care.

The rest of the paper is structured as follows: first our methods are set out, paying attention to our general approach, the input data that were needed to populate the model as well as the resource allocation model. Second, results are presented in terms of total costs and health benefits that may be obtained from the optimal allocation of a given budget. Finally we discuss the results and their policy implications.

## Methods

### General approach

To analyze the trade-off between four types of interventions for the general population and in diabetes patients, the following steps were taken. First, effects of the interventions on intermediate outcomes and intervention costs were estimated. Second, modelling was applied to find long term health effects and effects on healthcare costs, using the same model for all interventions. Third, capacity constraints and demand restrictions that may apply to the interventions were assessed. Fourth, the long term costs and effects were fed into a mathematical programming model, combining them with information on constraints and on population sizes to find optimal allocations for a range of healthcare budgets.

### Input data

Details and results of the first two steps have been published for all interventions concerned in separate publications [27-32]. In short, first interventions were selected based on the available evidence on effectiveness from systematic reviews and their relevance for the Dutch setting. For these interventions effects of the interventions on intermediate outcomes were estimated based on systematic reviews, while intervention costs were calculated using bottom up estimates of resource use and unit costs (Table 1). Cost data are expressed in euro at price levels 2007.

**Table 1:** Short term costs and effects of interventions (price level 2007)

Intervention	Effectiveness*	**Annual costs per participant**^†^
*General population*		

Minimal cessation counseling by GP	28	€30

Intensive smoking cessation counseling plus pharmacotherapy	68	€420

Minimal lifestyle intervention, community intervention (Hartslag Limburg^‡^)	Activity: 0-1 Overweight: 5-8	€6

Intensive lifestyle intervention for persons with extreme overweight (SLIM^§^)	Activity: 1-6 Overweight: 18.	€700

Medication to reduce blood pressure for persons with SBP > 140	390	€1200-€280**

Statins for persons with total cholesterol > 6.5	470	€1500-€3700**

*Diabetes patients*		

Minimal cessation counseling by GP	28	€30

Intensive smoking cessation counseling plus pharmacotherapy	68	€420

Minimal lifestyle intervention (X-PERT^††^)	Activity: 50-90 Overweight: 35	€120

Intensive lifestyle intervention (LookAHEAD^‡‡^)	Overweight: 140	€500

Medication to reduce blood pressure for persons with SBP > 140^§§^	390	€1000-€3300**

Statins for al diabetes patients***	470	€1100-€3800 **

* Short term effects expressed as the number of additional persons per 1000 participants that quit smoking, loose weight, increase activity, or continue lifelong medication. Only continuous drug use was assumed to lead to effects on disease risks, the latter were different for the general population and diabetes patients and for age and baseline risk [30,32]. Long term effects were age dependent and computed using the RIVM-Chronic Disease Model.

^† ^Intervention costs only. Effects on costs of care were age dependent and computed in the RIVM-Chronic Disease Model. Earlier publications provide more details on the intervention cost estimates [27-32]. All estimates were adjusted to price level 2007 using consumer price indices.

^‡ ^Ronkers et al. [34]

^§ ^Mensink et al. [35]

** Costs of lifetime medication use and consults were age dependent.

^†† ^Deakin et al. [36]

^‡‡ ^Pi-Sunyer et al. [37]

^§§ ^Effects given are the number of persons that continue lifetime medication. Effects of medication on disease risks were based on a meta-analysis [38]. For full details see the RIVM report by Jacobs-van der Bruggen et al. 2007 (available at http://www.rivm.nl/bibliotheek/rapporten/260801004.pdf).

*** Effects given are the number of persons that continue lifetime medication. Effects of medication on disease risks were based on a meta-analysis [6]. For full details see Jacobs-van der Bruggen et al. 2008 [30] and the RIVM report mentioned above.

The interventions in the general population are in principle also available for people with diabetes. However, it was assumed that the diabetes specific interventions get priority in case of overlap of target groups. Costs for the medication interventions specific for diabetes patients may differ from similar interventions in the general population, the main reason being different brands of medications typically used and cost sharing with other diabetes control consults. Effects and costs of interventions were corrected for relapse and non-adherence. For smoking, relapse was extensively modelled [33], while for overweight and activity, the effect of relapse was included in the final estimate of effectiveness [29,31]. For cholesterol lowering drugs and blood pressure control medication, a correction for non-adherence was done for those that would cease medication use within two years by excluding health gains and drug related costs after these two years [30,32].

### Simulation model

The RIVM Chronic Disease Model (CDM) and its diabetes module were applied to compute the long term effects of the interventions. The CDM is a Markov-type simulation model,[20] and comprises epidemiological data quantifying associations between multiple risk factors and chronic diseases among which cardiovascular diseases and cancers. The CDM diabetes module simulates the Dutch diabetes population [31]. The CDM has been used among others to evaluate long-term outcomes for diabetes prevention and treatment [29-31].

Current practice in the Netherlands served as a benchmark case, so that costs and effects are to be interpreted as additional values compared to current practice. Net cumulative gains in (quality adjusted) life expectancy and net effects on the present value of health care costs were estimated over a lifetime horizon. Costs and effects were tracked until the last person of the cohort had died, for 3 age groups, 20-44 years, 45-64 years and 65 years and older. Outcomes in future years were discounted at the rates prescribed by the current Dutch guidelines for pharmacoeconomic evaluations (4% and 1.5% annually for health effects and costs respectively.) Total costs per QALY for all 12 interventions, for three age categories, for the intervention compared to usual care were estimated (cf Table 1).

### Constraints

In a third step, capacity and demand constraints were added. For each age group and risk factor, the total number of persons receiving an intervention cannot be more than the total size of the target population. For instance, it is impossible to offer more smoking cessation support courses for 65 and over than the number of smokers at that ages. This results in a set of restrictions that were added to the basic optimization model. Their values were specified for the three age categories in Table S1 (Additional file 1) and were derived from information about lifestyle in the Dutch (diabetes) population and availability of treatments. Furthermore, for each intervention, constraints were added to reflect that the total number of participants over all age groups for each intervention was limited by professional capacity. These restrictions will be referred to as capacity constraints (see Additional file 1, Table S1).

### Optimization model

The optimization model used in current application may now be formally written as follows.

(1) $\underset{p^{ja}}{\mathrm{Max}}\;\underset{j}{\sum }\underset{a}{\sum }p^{ja}q^{ja}$ subject to

(2) $\underset{j}{\sum }\underset{a}{\sum }p^{ja}c^{ja}\leq b$ and *b *given.

(3) $\underset{a}{\sum }p^{ja}\leq cap^{j}$, for all j,

(4) 0 ≤ *p^ja ^*≤ *dem^ja^*, for all j, for all a

With:

j      Index for programs, j = 1,...12.

a      Index for age, a = 1,..3 age groups distinguished

*p^ja ^*   Number of people of age a receiving program j

b      Total available budget (Net present value over entire time horizon)

*q ^ja ^*   Health effects per participant of program j for people of age a (Net present value)

*c ^ja ^*   Costs of program j per participant of age a (Net present value)

*dem^ja ^*   Demand restrictions for program j and age group a

*cap^j ^*   Capacity constraints for program j

The simulation model provided estimates for the health effects and costs per participant (q ^ja ^and c ^ja^). These were combined with relevant constraints to form the resource allocation model, which was then solved using the linear programming features of Mathematica.(routine LinearProgramming)

Constraints for demand were assumed to be age group specific, while capacity constraints were given for each program over all age groups together.

### Sensitivity analyses

The standard model was analyzed for a range of different budgets, to find optimal combinations of total health and total costs. Then, we removed the capacity constraints to estimate their effect in a second analysis. Finally, sensitivity analyses investigated the robustness of the results for different discount rates and time horizons.

## Results

### Cost-effectiveness ratios

Table 2 shows the interventions in the different age categories ordered at increasing costs per QALY. For most interventions, long term cost effectiveness was lowest for the lowest age category, since at this age the full effects of prevention could be included, before any harm has been done. The exceptions were statins for diabetes patients and blood pressure treatment for the general population, reflecting that for this age category too many unnecessary cases will be treated lifelong.

**Table 2:** Costs per QALY compared to care as usual

Average costs per QALY (euro)	Age category	Target population	Intervention (Short name)
1400	20-44	General population	Minimal cessation counseling by GP (S1)

1500	20-44	Diabetes patients	Minimal cessation counseling by GP (Sd1)

2700	45-64	Diabetes patients	Minimal cessation counseling by GP (Sd2)

2900	45-64	General population	Minimal cessation counseling by GP (S2)

3000	20-44	General population	Hartslag Limburg (HL1)

5400	45-64	General population	Hartslag Limburg (HL2)

5800	20-44	Diabetes patients	LookAHEAD (LA1)

5900	20-44	Diabetes patients	X-PERT (XP1)

6400	20-44	Diabetes patients	Intensive smoking cessation counseling plus pharmacotherapy((ISd1)

6700	20-44	General population	Intensive smoking cessation counseling plus pharmacotherapy (IS1)

6800	20-44	Diabetes patients	Medication to reduce blood pressure for persons with SBP > 140 (BPd1)

7400	45-64	Diabetes patients	X-PERT (XP2)

7800	45-64	Diabetes patients	Medication to reduce blood pressure for persons with SBP > 140 (BPd2)

8000	65+	Diabetes patients	Minimal cessation counseling by GP (Sd3)

8600	45-64	General population	Intensive smoking cessation counseling plus pharmacotherapy (IS2)

9200	45-64	Diabetes patients	Intensive smoking cessation counseling plus pharmacotherapy (ISd2)

9800	45-64	Diabetes patients	Statins for all diabetes patients (Std2)

10100	45-64	Diabetes patients	LookAHEAD (LA2)

10500	65+	General population	Minimal cessation counseling by GP (S3)

10900	45-64	General population	Medication to reduce blood pressure for persons with SBP > 140 (BP2)

11000	20-44	Diabetes patients	Statins for all diabetes patients (Std1)

11200	20-44	General population	Medication to reduce blood pressure for persons with SBP > 140 (BP1)

12900	65+	Diabetes patients	Medication to reduce blood pressure for persons with SBP > 140 (BPd3)

16100	65+	General population	Hartslag Limburg (HL3)

16600	65+	General population	Medication to reduce blood pressure for persons with SBP > 140 (BP3)

16600	65+	Diabetes patients	Statins for all diabetes patients (Std3)

18100	20-44	General population	Statins for persons with total cholesterol > 6.5 (St1)

18500	45-64	General population	Statins for persons with total cholesterol > 6.5 (St2)

19700	65+	Diabetes patients	X-PERT (XP3)

19900	20-44	General population	SLIM (SL1)

27300	45-64	General population	SLIM (SL2)

28100	65+	General population	Statins for persons with total cholesterol > 6.5 (St3)

32300	65+	Diabetes patients	Intensive smoking cessation counseling plus pharmacotherapy (ISd3)

33200	65+	Diabetes patients	LookAHEAD (LA3)

35500	65+	General population	Intensive counseling plus pharmacotherapy (IS3)

59600	65+	General population	SLIM (SL3)

For the interventions in each age category ordered at worsening cost-effectiveness. (Net present values over a lifetime horizon. Discount rates 4% for costs and 1.5% for QALYs, price level 2007.).

Based on these cost-effectiveness ratios only, low budgets would seem to be spent primarily in diabetes patients: In total 17 interventions had average cost-effectiveness below €10,000 per QALY, and 11 of these were for diabetes patients. The 13 interventions costing between €10,000 and €20,000 per QALY consisted of 5 diabetes interventions and 8 interventions for the general population. Finally, 6 interventions cost more than €20,000 per QALY, and 4 of these were for the general population. That is, interventions in the diabetes population were mostly more cost-effective than in the general population reflecting the effect of targeting to a high risk group. However, low intensity overweight and activity programs were more cost-effective in the general population. This may be explained from the relatively higher effectiveness of the general population program. It cost much less and had relatively a better effect. A possible explanation is that diabetes patients already experienced serious problems from being overweight and yet did not succeed in loosing weight, so they may need more intensive programs to successfully loose weight.

### Proportion of money spent in the general population

Table 3 shows optimal allocations and incremental cost-effectiveness ratios for a range of total budgets. At most these 12 interventions offered the possibility to gain an additional 560,000 QALY, for about €640 per capita in additional costs over the entire time horizon. Table 3 also presents the percentages of health gains and money obtained from prevention in the general population. At low budgets, all money was optimally allocated towards this type of interventions, especially smoking cessation and overweight reduction. Moderately high budgets however (that is, more than €9 per capita, or below €100 per capita), were spent mostly on prevention in diabetes. The optimal set now additionally included increased use of statins and medication for blood pressure in diabetes patients as well as intensive overweight reduction. Finally, for very high budgets, above €100 per capital, additional medication for the general population was added. The majority of budgets were again allocated to prevention in the general population. Hence, the optimal distribution of money between interventions in diabetes patients or the general population depended on available budgets.

**Table 3:** Optimization results for different budgets

Budget (€ *10^6)	Spent in general population (%)	Total health gains (QALY*1000)	Gained in general population (%)	Incremental costs per QALY	**Changes in interventions chosen **^**i**^
1	100	700	100	€1,400	+ S1

10	100	6,950	100	€1,400	NA^ii^

100	89	29,400	90	€6,700	+ IS1, Sd1, Sd2, HL1, HL2, XP1

250	65	50,400	74	€7,400	+ XP2, BPd1, BPd2

500	32	78,100	47	€9,800	+ Sd3, Std2

750	24	103,000	37	€10,900	+ ISd1, LA2, BP2 - Sd1

1,000	43	126,000	49	€10,900	NA

2500	77	264,000	76	€10,900	NA

5,000	83	440,000	81	€18,100	+ ISd2, BP1, BPd3, St1 - Sd2

7,253	88	561,000	84	€49,300	+ ISd3, SL1, LA3, St2, Std1 - Sd3

Maximal health gains and incremental costs per QALY for a range of different budgets. Net present values over a lifetime horizon. Discount rates 4% for costs and 1.5% for QALYs, price level 2007. Interventions added as compared to the set chosen for the budget in the previous row are indicated by +, interventions removed are indicated by -.

^i ^A list of the interventions and their abbreviations is given in Table 2.

^ii ^That is, more money was spent on the same set of interventions as in the previous row

From Table 2 ranking on cost-effectiveness ratios only seemed to indicate that targeted prevention was more efficient in general. However, the optimal allocations (Table 3) showed that due to varying sizes of target populations and capacity constraints no general a priori priority for either type of prevention existed and it depended on the size of the budget, as well as available interventions, whether most resources were spent in universal prevention or in targeted prevention or both.

### Effects of supply limits

Running the optimization model without the capacity constraints on the maximal supply of each intervention resulted in almost a doubling of maximal potential health gains from 0.56 million QALY to 0.96 million QALY. Table 4 below gives the outcomes of a model without capacity limits, for the same range of budgets as in Table 3. The maximal total budget to be spent on the 12 interventions was of course higher and amounted to circa €1200 per capita.

**Table 4:** Optimization results in model without capacity constraints

Budget (€ *10^6)	Spent in general population (%)	Total health gains (QALY *1000)	Gained in general population (%)	Incremental costs per QALY
1	100	700	100	€1,460

10	100	6,950	100	€1,460

100	96	49,600	96	€3,040

250	96	78,000	96	€7,230

500	74	113,000	84	€8,510

750	73	142,000	81	€10,900

1000	55	168,000	68	€10,900

2500	70	309,000	73	€13,700

5000	77	516,000	78	€20,000

7250	78	651,000	78	€20,800

10,000	84	801,000	82	€21,700

Maximal budget: 13,587	87	958,000	84	€426,000

Maximal health gains and incremental costs per QALY for a range of different budgets. Model without capacity constraints. (Net present values over a lifetime horizon. Discounted rates 4% for costs and 1.5% for QALYs, price level 2007.)

**Figure 1:**
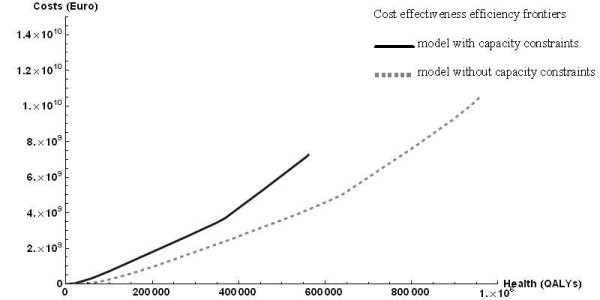
**Cost effectiveness efficiency frontiers**. model with capacity constraints (dark, solid line). model without capacity constraints (light, dashed line).

Figure 1 shows optimal combinations of budgets and total health effects, that is, the choices that obtain most health for a given budget. The steepness of the different line segments represents the incremental cost-effectiveness ratios. In other words, they reflect the additional costs that must be paid for one additional QALY, if the extra money is spent in the most efficient way. At corner points, one or more constraints force a change in the set of programs chosen. The two lines represent the model with and without capacity constraints and illustrate the effects of these constraints. For any given budget, less health can be obtained, while the upper limit to health benefits is substantially reduced in case of capacity constraints.

Comparison of Tables 3 and 4 shows that the capacity constraints reduced the percentage of the budget spent in the general population. This indicates that capacity constraints were more limiting for interventions in the general population than for interventions targeted at diabetes patients.

### Sensitivity analyses

**Figure 2:**
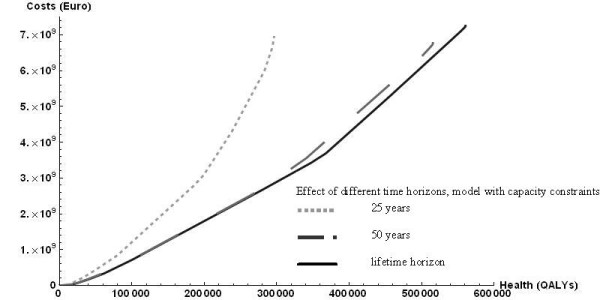
**Effect of different time horizons, model with capacity constraints**. 25 years (light dotted line). 50 years (grey dashed line). lifetime horizon (black solid line, reference case).

Sensitivity analyses showed that the time horizon mattered, because at shorter time horizons, neither the full costs in life years gained, nor the full health effects could be realized. Thus, maximal total costs and maximal total health effects were smaller. Figure 2 shows the efficiency frontiers for time horizons of 25, and 50 years, compared to the lifetime horizon chosen in the main analysis. Too short time horizons caused relevant effects to be left out of the analysis.

Furthermore, the outcomes were sensitive to the rate of discount, as is usually the case in economic evaluations of prevention, with health outcomes occurring far in the future and intervention costs having to be paid immediately. The base case discounted health effects at 1.5% and costs at 4%, which is the current Dutch standard (cf http://www.cvz.nl). For discount rates at 4% for both health and costs, the efficiency frontier moved inward, since the net present value of health effects decreased. For discount rates of 0% on both health and costs, it moved outward. Increasing the difference in discounting between costs and health effects, with health effects discounted at 0%, rather than 1.5%, moved the efficiency frontier outward.

## Discussion

The current study used a resource allocation model to analyze prevention of diabetes and its complications in the Netherlands. Optimal resource allocations were computed over a set of 12 interventions aiming to reduce the risk for diabetes and/or cardiovascular disease, either in the general population or in diabetes patients. While for small and high budgets the majority of money would go to interventions in the general population, moderately high budgets were mostly spent in diabetes patients.

Strengths of the resource allocation approach were that it was relatively straightforward to account for constraints and analyze their effects. These constraints are for instance due to limited capacity to provide interventions. Removing constraints on intervention supply increased maximal additional expenditure from €640 to €1200 per capita and almost doubled maximal potential health gains. The constraints were more limiting for prevention in the general population than for interventions in diabetes patients. This makes sense, since the group of diabetes patients is much smaller.

The model used to evaluate long term health effects took into account limited effectiveness and adherence, competing risks, and relapse. Hence, the estimates took care not to overestimate health effects. Our special attention went to the health care costs to be included in the budget allocation model. In this study, costs consisted of intervention costs plus the full long term effects of prevention on health care costs. Alternatively only intervention costs could be included in the budgets. While the latter may result in numbers that are closer to common sense ideas about the sizes of the budgets at stake, it is inconsistent from a long term perspective [21]. Changing to short term budgets increased the variability of choices between prevention in the general population and targeted prevention (results not shown).

Another distinctive feature of our modeling exercise is that we accounted for quality of life decreases with advancing age. This is important, since obviously most of the life years gained occur at advanced ages.

While the current results were specific for the Netherlands, the general approach could be applied to any setting. This would require either an existing disease model comparable to the RIVM chronic disease model, or a transfer of this model to the appropriate setting, replacing prevalence, incidence and mortality parameters by setting specific estimates. Furthermore, the cost estimates of the interventions, as well as the estimates of capacities and further constraints should be adjusted if they were expected to differ from the Dutch estimates.

Similar recent applications of resource allocation in diabetes and in obesity prevention have appeared in the UK and in Australia [23,26]. The study by Segal focused on prevention in the general population, especially different types of overweight control. Indirect medical costs were not included and costs were computed per life year gained, ignoring effects on quality of life. The study by Earnshaw only considered prevention in the diabetes population. In contrast, the current study also included interventions in the general population and therefore allowed to explore the trade off between both types of prevention. Furthermore, Earnshaw used a full experimental design to directly compute results for any combination of prevention interventions. In the current paper, a simpler approach was applied with only single intervention policies modeled, assuming additive health effects. Third, Earnshaw focused on intervention costs only, which implies the implicit assumption that health care cost effects would be the same for all interventions. That is clearly not the case for interventions on overweight versus smoking cessation or statin treatment. Finally, they did not incorporate age effects on quality of life, which is important if trade-offs are made between age groups.

While a number of diabetes models have been published in recent years, [39] for the current application we preferred to use the RIVM Chronic Disease Model (CDM). While this model maybe less well known, all parameters estimates are accessible and the general structure of the CDM as well as relevant applications have been published in peer reviewed journals [20,27-33]. The most important advantage of this model for our current purpose was that it allows evaluating interventions in the general population and in diabetes patients using the same model.

Some assumptions in our current study require further discussion. First of all, combinations of interventions were assumed to have no specific interaction effects, that is, the health gains in terms of life years and QALYs gained were assumed additive. This same assumption was made for instance in the global burden of disease study [40]. It probably implies an overestimation of total health effects if persons receive more than one intervention. This assumption is a bit more problematic in the diabetes population than in the general population. Thus the effects of the diabetes interventions may have been overestimated as compared to interventions in the general population, implying that the optimal shares of money spent in the general population might be higher than our results indicated. Second, another assumption applied in the current paper was the possibility to offer interventions to a population of variable size, by varying the budget spent on each intervention. Some resource allocation models pay specific attention to the consequences of having indivisible interventions of fixed sizes [41]. The optimization problem then changes into a so called integer programming problem. The question whether program size is variable or not depends on the interventions at stake. For the current interventions, it was rather easy to vary sizes by having more or less money available for them, because most of them were supply driven and addressed people that are not yet acutely ill. For curative interventions, varying program size may be more problematic, since it would imply that some actual patients would receive improper treatment.

While we did provide sensitivity analyses for the effects of discount rates, time horizon and budgetary constraints, a more extensive uncertainty analysis would improve insight into the robustness of our outcomes. This requires the use of stochastic programming techniques and we would like to address this issue in future research.

A further advantage of the resource allocation approach is that once the model has been formulated, it is easy to vary constraints and objectives, for instance on indivisible programs or equity [23,24]. The current results on capacity constraints might help to focus efforts to extend prevention capacity to those areas where it would be most worthwhile, using the shadow prices of the constraints.

A drawback of resource allocation models may be seen in their data greediness. However, most of these data would be needed for careful priority setting anyway. The only additional requirement for a budget allocation model is that all data used are consistent and can be sensibly combined in the same model. Therefore, using a resource allocation model forces to seek for consistent, well comparable data, and that maybe considered an advantage rather than a drawback [17].

## Conclusions

Resource allocation models may help health care decision makers to integrate information about the costs, sizes, and health effects of sets of programs. Our diabetes application had U-shaped results: prevention in the general population was the best way to retain health benefits for low and high budgets, while moderate budgets would mostly be spent on prevention in diabetes patients. Targeted prevention in diagnosed patients was therefore not a priori more or less efficient than prevention in the general population. The application also showed that an additional 560 thousand QALYs may be gained by currently available interventions even when accounting for existing capacity and demand limits.

## Competing interests

The authors declare that they have no competing interests.

## Authors' contributions

TF and CB initiated the research/got funding. TF and PvB developed the BA model. GJK, PvB and TF wrote code and did analyses. TF, MJ and PvB gathered the input data and evaluated interventions with the RIVM CZM. RH developed the RIVM CZM, PvB and RH developed the RIVM CZM+diabetes module as applied in this study. TF and PvB wrote the first draft article. All authors contributed to important revisions and read and approved the final manuscript. CB acts as a guarantor for the project.

## Supplementary Material

Additional file 1 **Table S1**. Table with information about constraints on the demand and capacity of interventions. Click here for file

## References

[B1] Hamman RF Wing RR Edelstein SL Lachin JM Bray GA Delahanty L Hoskin M Kriska AM Mayer-Davis EJ Pi-Sunyer X Regensteiner J Venditti B Wylie-Rosett J Effect of weight loss with lifestyle intervention on risk of diabetes Diabetes Care 2006 29 9 2102 2107 10.2337/dc06-0560 16936160 PMC1762038

[B2] Hartemink N Boshuizen HC Nagelkerke NJ Jacobs MA van Houwelingen HC Combining risk estimates from observational studies with different exposure cutpoints: a meta-analysis on body mass index and diabetes type 2 Am J Epidemiol 2006 163 11 1042 1052 10.1093/aje/kwj141 16611666

[B3] Chaturvedi N The burden of diabetes and its complications: trends and implications for intervention Diabetes Res Clin Pract 2007 76 Suppl 1 S3 12 17343954 10.1016/j.diabres.2007.01.019

[B4] Mainous AG Baker R Koopman RJ Saxena S Diaz VA Everett CJ Majeed A Impact of the population at risk of diabetes on projections of diabetes burden in the United States: an epidemic on the way Diabetologia 2007 50 5 934 940 10.1007/s00125-006-0528-5 17119914 PMC1849422

[B5] Redekop WK Koopmanschap MA Rutten GE Wolffenbuttel BH Stolk RP Niessen LW Resource consumption and costs in Dutch patients with type 2 diabetes mellitus. Results from 29 general practices Diabet Med 2002 19 3 246 253 10.1046/j.1464-5491.2002.00654.x 11918627

[B6] Baigent C Keech A Kearney PM Blackwell L Buck G Pollicino C Kirby A Sourjina T Peto R Collins R Simes R Efficacy and safety of cholesterol-lowering treatment: prospective meta-analysis of data from 90,056 participants in 14 randomised trials of statins Lancet 2005 366 9493 1267 1278 16214597 10.1016/S0140-6736(05)67394-1

[B7] Gaede P Vedel P Larsen N Jensen GV Parving HH Pedersen O Multifactorial intervention and cardiovascular disease in patients with type 2 diabetes N Engl J Med 2003 348 5 383 393 10.1056/NEJMoa021778 12556541

[B8] Marshall T Rouse A Resource implications and health benefits of primary prevention strategies for cardiovascular disease in people aged 30 to 74: mathematical modelling study BMJ 2002 325 7357 197 10.1136/bmj.325.7357.197 12142309 PMC117452

[B9] Bottomley JM Raymond FD Pharmaco-economic issues for diabetes therapy Best Pract Res Clin Endocrinol Metab 2007 21 4 657 685 10.1016/j.beem.2007.08.002 18054741

[B10] Vijgen SMC Hoogendoorn M Baan CA de Wit GA Limburg W Feenstra TL Cost effectiveness of preventive interventions in type 2 diabetes mellitus: a systematic literature review Pharmacoeconomics 2006 24 5 425 441 10.2165/00019053-200624050-00002 16706569

[B11] Avenell A Broom J Brown TJ Poobalan A Aucott L Stearns SC Smith WC Jung RT Campbell MK Grant AM Systematic review of the long-term effects and economic consequences of treatments for obesity and implications for health improvement Health Technol Assess 2004 8 21 182 10.3310/hta8210 15147610

[B12] Franco OH Peeters A Looman CW Bonneux L Cost effectiveness of statins in coronary heart disease J Epidemiol Community Health 2005 59 11 927 933 10.1136/jech.2005.034900 16234419 PMC1732951

[B13] Gordon L Graves N Hawkes A Eakin E A review of the cost-effectiveness of face-to-face behavioural interventions for smoking, physical activity, diet and alcohol Chronic Illn 2007 3 2 101 129 10.1177/1742395307081732 18083667

[B14] Shearer J Shanahan M Cost effectiveness analysis of smoking cessation interventions Aust N Z J Public Health 2006 30 5 428 434 10.1111/j.1467-842X.2006.tb00458.x 17073223

[B15] Ward S Lloyd Jones M Pandor A Holmes M Ara R Ryan A Yeo W Payne N A systematic review and economic evaluation of statins for the prevention of coronary events Health Technol Assess 2007 11 14 1 iv 17408535 10.3310/hta11140

[B16] Gray AM Clarke P The economic analyses of the UK prospective diabetes study Diabet Med 2008 25 Suppl 2 47 51 18717979 10.1111/j.1464-5491.2008.02502.x

[B17] Philips Z Bojke L Sculpher M Claxton K Golder S Good practice guidelines for decision-analytic modelling in health technology assessment: a review and consolidation of quality assessment Pharmacoeconomics 2006 24 4 355 371 10.2165/00019053-200624040-00006 16605282

[B18] Pang F Design, analysis and presentation of multinational economic studies: the need for guidance Pharmacoeconomics 2002 20 2 75 90 10.2165/00019053-200220020-00001 11888360

[B19] Welte R Feenstra T Jager H Leidl R A decision chart for assessing and improving the transferability of economic evaluation results between countries Pharmacoeconomics 2004 22 13 857 876 10.2165/00019053-200422130-00004 15329031

[B20] Hoogenveen RT van Baal PH Boshuizen HC Chronic disease projections in heterogeneous ageing populations: approximating multi-state models of joint distributions by modelling marginal distributions Math Med Biol 2010 27 1 1 19 10.1093/imammb/dqp014 19516046

[B21] Van Baal PHM Feenstra TL Hoogenveen RT De Wit GA Brouwer WBF Unrelated medical care in life years gained and the cost utility of primary prevention: in search of a 'perfect' cost-utility ratio Health Econ 2007 16 4 421 433 10.1002/hec.1181 17039573

[B22] Gafni A Birch S Incremental cost-effectiveness ratios (ICERs): the silence of the lambda Soc Sci Med 2006 62 9 2091 2100 10.1016/j.socscimed.2005.10.023 16325975

[B23] Earnshaw SR Richter A Sorensen SW Hoerger TJ Hicks KA Engelgau M Thompson T Narayan KM Williamson DF Gregg E Zhang P Optimal allocation of resources across four interventions for type 2 diabetes Med Decis Making 2002 22 5, Suppl S80 S91 10.1177/027298902237704 12369234

[B24] Epstein DM Chalabi Z Claxton K Sculpher M Efficiency, equity, and budgetary policies: informing decisions using mathematical programming Med Decis Making 2007 27 2 128 137 10.1177/0272989X06297396 17409363

[B25] Niessen LW Dijkstra R Hutubessy R Rutten GE Casparie AF Lifetime health effects and costs of diabetes treatment Neth J Med 2003 61 11 355 364 14768718

[B26] Segal L Dalton AC Richardson J Cost-effectiveness of the primary prevention of non-insulin dependent diabetes mellitus Health Promotion International 1998 13 3 197 209 10.1093/heapro/13.3.197

[B27] Bemelmans W van BP Wendel-Vos W Schuit J Feskens E Ament A Hoogenveen R The costs, effects and cost-effectiveness of counteracting overweight on a population level. A scientific base for policy targets for the Dutch national plan for action Prev Med 2008 46 2 127 132 10.1016/j.ypmed.2007.07.029 17822752

[B28] Feenstra TL Hamberg-van Reenen HH Hoogenveen RT Rutten-van Molken MPMH Cost-effectiveness of face-to-face smoking cessation interventions: a dynamic modeling study Value Health 2005 8 3 178 190 10.1111/j.1524-4733.2005.04008.x 15877590

[B29] Jacobs-van der Bos G Bemelmans WJ Hoogenveen RT Vijgen SM Baan CA Lifestyle interventions are cost-effective in people with different levels of diabetes risk: results from a modeling study Diabetes Care 2007 30 1 128 134 10.2337/dc06-0690 17192345

[B30] Jacobs-van der Engelfriet PM Hoogenveen RT van Baal PH Struijs JN Verschuren WM Smit HA Baan CA Lipid-lowering treatment for all could substantially reduce the burden of macrovascular complications of diabetes patients in the Netherlands Eur J Cardiovasc Prev Rehabil 2008 15 5 521 525 10.1097/HJR.0b013e3283041523 18830084

[B31] Jacobs-van der Bruggen MAM van Baal PH Hoogenveen RT Feenstra TL Briggs AH Lawson K Feskens EJM Baan CA Cost-effectiveness of lifestyle modification in diabetes patients Diabetes Care 2009 32 8 1453 1458 10.2337/dc09-0363 19435958 PMC2713648

[B32] Kok L Engelfriet P Jacobs-van der Hoogenveen RT Boshuizen HC Verschuren MW The cost-effectiveness of implementing a new guideline for cardiovascular risk management in primary care in the Netherlands Eur J Cardiovasc Prev Rehabil 2009 16 3 371 376 10.1097/HJR.0b013e328329497a 19305351

[B33] Hoogenveen RT van Baal PH Boshuizen HC Feenstra TL Dynamic effects of smoking cessation on disease incidence, mortality and quality of life: the role of time since cessation Cost Eff Resour Alloc 2008 6 1 10.1186/1478-7547-6-1 PMC2267164 18190684

[B34] Ronckers ET Groot W Steenbakkers M Ruland E Ament A Costs of the 'Hartslag Limburg' community heart health intervention BMC Public Health 2006 6 no51 10.1186/1471-2458-6-51 PMC1450273 16512909

[B35] Mensink M Blaak EE Corpeleijn E Saris WH de Bruin TW Feskens EJ Lifestyle intervention according to general recommendations improves glucose tolerance Obes Res 2003 11 12 1588 96 10.1038/oby.2003.211 14694225

[B36] Deakin TA Cade JE Williams R Greenwood DC Structured patient education: the diabetes X-PERT Programme makes a difference Diabet Med 2006 23 9 944 954 10.1111/j.1464-5491.2006.01906.x 16922700

[B37] Pi-Sunyer X Blackburn G Brancati FL Bray GA Bright R Clark JM Curtis JM Espeland MA Foreyt JP Graves K Haffner SM Harrison B Hill JO Horton ES Jakicic J Jeffery RW Johnson KC Kahn S Kelley DE Kitabchi AE Knowler WC Lewis CE Maschak-Carey BJ Montgomery B Nathan DM Patricio J Peters A Redmon JB Reeves RS Ryan DH Safford M Reduction in weight and cardiovascular disease risk factors in individuals with type 2 diabetes: one-year results of the look AHEAD trial Diabetes Care 2007 30 6 1374 1383 17363746 10.2337/dc07-0048 PMC2665929

[B38] Turnbull F Neal B Algert C Chalmers J Chapman N Cutler J Woodward M MacMahon S Effects of different blood-pressure lowering regimens on major cardiovascular events in individuals with and without diabetes mellitus: results of prospectively designed overviews of randomized trials Arch Intern Med 2005 165 1410 1419 15983291 10.1001/archinte.165.12.1410

[B39] Mount Hood 4 modeling group Computer modeling of diabetes and its complications: a report on the Fourth Mount Hood Challenge Meeting Diabetes Care 2007 30 6 1638 1646 17526823 10.2337/dc07-9919

[B40] Murray CJL Lopez AD Jamison DT The global burden of disease in 1990: summary results, sensitivity analysis and future directions Bull World Health Organ 1994 72 3 495 509 8062404 PMC2486716

[B41] Sendi P Al MJ Gafni A Birch S Optimizing a portfolio of health care programs in the presence of uncertainty and constrained resources Soc Sci Med 2003 57 11 2207 2215 10.1016/S0277-9536(03)00086-8 14512250

