# Supplementary material for: Targeted versus universal prevention. a resource allocation model to prioritize cardiovascular prevention
Source: Cost Eff Resour Alloc. 2011 Oct 6;9:14. doi: 10.1186/1478-7547-9-14 (PMC3200148; doi:10.1186/1478-7547-9-14)
Supplement: Additional file 1 — Table S1. Table with information about constraints on the demand and capacity of interventions. [file 1478-7547-9-14-S1.DOC]

Additional File 1

Table with constraints on the demand and capacity of interventions

Table S1: Constraints on demand and capacity of interventions, as maximal numbers of participants

| Intervention | Demand constraint (*1000)  [age groups  25-44;45-64;65+] | Capacity constraint ^i^  (*1000) |
| --- | --- | --- |
| *General population* | | |
| Minimal cessation counseling by GP | 1100;770;190 **, ^a^ | 445 |
| Intensive smoking cessation counseling plus pharmacotherapy | 630 ;440;110 **, ^b^ | 260 |
| Community intervention aimed at weight reduction and a healthy lifestyle (Hartslag Limburg) | 1880;2040;1100 ^c^ | 2400 |
| Diet and exercise, high intensity intervention for persons with extreme overweight (SLIM) | 200 ;250;120 ^d^ | 200 |
| Medication to reduce blood pressure for persons with SBP>140 | 290;780;950 | 1065 |
| Statins for persons with cholesterol>6.5 | 460;1010;580 | 1065 |
| *Diabetes patients* | | |
| Minimal cessation counseling by GP | 20;70;60 **^,e^ | Not applicable (na) |
| Intensive cessation counseling plus pharmacotherapy | 20;70;60 **^,e^ | na |
| Minimal lifestyle intervention (X-PERT) | 40;200;300 | 130 |
| Intensive lifestyle intervention (LookAHEAD) | 0;120;180 | 130 |
| Medication to reduce blood pressure for persons with SBP >140 | 2;40;130 | 550 |
| Statins | 40;180;270 | 120 |

**All demand figures for smokers were corrected for 15% of smokers that never wants to quit (Stivoro, 2007).

^a^ Smokers visiting their GP at least once in the current year (Vijgen, 2007). ^b^Smokers with at least one hospital outpatient visit in a year.(Vijgen, 2007) ^c^ It was assumed that 90% of a community would participate. ^d^ It was assumed that 50% of people with obesity would participate. ^e^ all smoking diabetes patients have at least one annual contact with a health care profession by assumption. ^i^ Based on time and availability of professionals (Vijgen 2007; Bemelmans 2008; Kok 2009; Jacobs 2007). For diabetes patients, capacity constraints did not apply to the smoking cessation interventions that were similar to their general population counterparts. For the medication interventions, capacity constraints were based on estimated numbers of people qualifying for this medication based on their risk profile and a realistic scenario for statin use in diabetes.

*References*

Vijgen SMC, Gelder BM van, Baal PHM van, Zutphen M van, Hoogenveen RT, Feenstra TL (2007) Cost and effects of tobacco cessation. RIVM report no 260601004, RIVM, Bilthoven, The Netherlands. (available at www.rivm.nl)

Bemelmans W, van Baal P, Wendel-Vos W, et al. (2008) The costs, effects and cost-effectiveness of counteracting overweight on a population level. A scientific base for policy targets for the Dutch national plan for action. Prev.Med. 46(2): 127-132.

Kok L, Engelfriet P, Jacobs-van der Bruggen MA, et al. (2009) The cost-effectiveness of implementing a new guideline for cardiovascular risk management in primary care in the Netherlands. Eur. J. Cardiovasc. Prev. Rehabil. 16(3): 371-376.

Jacobs-van der Bruggen MA, Bos G, Bemelmans WJ, et al. (2007) Lifestyle interventions are cost-effective in people with different levels of diabetes risk: results from a modeling study, Diabetes Care. 30(1): 128-134.
